# Exploring the effect of previous inactivated influenza vaccination on seasonal influenza vaccine effectiveness against medically attended influenza: Results of the European I‐MOVE multicentre test‐negative case‐control study, 2011/2012‐2016/2017

**DOI:** 10.1111/irv.12562

**Published:** 2018-05-09

**Authors:** Marta Valenciano, Esther Kissling, Amparo Larrauri, Baltazar Nunes, Daniela Pitigoi, Joan O'Donnell, Annicka Reuss, Judit Krisztina Horváth, Iwona Paradowska‐Stankiewicz, Caterina Rizzo, Alessandra Falchi, Isabelle Daviaud, Mia Brytting, Adam Meijer, Bernard Kaic, Alin Gherasim, Ausenda Machado, Alina Ivanciuc, Lisa Domegan, Brunhilde Schweiger, Annamária Ferenczi, Monika Korczyńska, Antonino Bella, Ana‐Maria Vilcu, Anne Mosnier, Katherina Zakikhany, Marit de Lange, Sanja Kurečić Filipovićović, Kari Johansen, Alain Moren, I‐MOVE primary care multicentre case‐control team

**Affiliations:** ^1^ Epidemiology Epiconcept Paris France; ^2^ National Centre of Epidemiology Institute of Health Carlos III Madrid Spain; ^3^ Department of Epidemiology Instituto Nacional de Saúde, Doctor Ricardo Jorge Lisboa Portugal; ^4^ University of Medicine and Pharmacy Carol Davila Bucharest Romania; ^5^ Cantacuzino Institute National Institute of Research – Development for Microbiology and Immunology Bucharest Romania; ^6^ Health Service Executive – Health Protection Surveillance Centre Dublin Ireland; ^7^ Department for Infectious Disease Epidemiology Robert Koch Institute Berlin Germany; ^8^ Department of Disease Prevention and Surveillance National Centre for Epidemiology Budapest Hungary; ^9^ National Institute of Public Health – National Institute of Hygiene Warsaw Poland; ^10^ National Center for Epidemiology, Surveillance and Health Promotion Istituto Superiore di Sanità Rome Italy; ^11^ Université de Corse‐Inserm Corte France; ^12^ Open Rome, Réseau des GROG Paris France; ^13^ The Public Health Agency of Sweden Stockholm Sweden; ^14^ Centre for Infectious Disease Control National Institute of Public Health and Environment (RIVM) Bilthoven The Netherlands; ^15^ Hrvatski zavod za javno zdravstvo Zagreb Croatia; ^16^ Institut Pierre Louis d'épidémiologie et de Santé Publique (IPLESP UMRS 1136) UPMC Univ Paris 06, INSERM Sorbonne Universités Paris France; ^17^ European Centre for Disease Prevention and Control (ECDC) Stockholm Sweden

**Keywords:** case‐control study, influenza, influenza vaccine, multicentre study, vaccine effectiveness

## Abstract

**Background:**

Results of previous influenza vaccination effects on current season influenza vaccine effectiveness (VE) are inconsistent.

**Objectives:**

To explore previous influenza vaccination effects on current season VE among population targeted for vaccination.

**Methods:**

We used 2011/2012 to 2016/2017 I‐MOVE primary care multicentre test‐negative data. For each season, we compared current season adjusted VE (aVE) between individuals vaccinated and unvaccinated in previous season. Using unvaccinated in both seasons as a reference, we then compared aVE between vaccinated in both seasons, current only, and previous only.

**Results:**

We included 941, 2645 and 959 influenza‐like illness patients positive for influenza A(H1N1)pdm09, A(H3N2) and B, respectively, and 5532 controls. In 2011/2012, 2014/2015 and 2016/2017, A(H3N2) aVE point estimates among those vaccinated in previous season were −68%, −21% and −19%, respectively; among unvaccinated in previous season, these were 33%, 48% and 46%, respectively (aVE not computable for influenza A(H1N1)pdm09 and B). Compared to current season vaccination only, VE for both seasons' vaccination was (i) similar in two of four seasons for A(H3N2) (absolute difference [ad] 6% and 8%); (ii) lower in three of four seasons for influenza A(H1N1)pdm09 (ad 18%, 26% and 29%), in two seasons for influenza A(H3N2) (ad 27% and 39%) and in two of three seasons for influenza B (ad 26% and 37%); (iii) higher in one season for influenza A(H1N1)pdm09 (ad 20%) and influenza B (ad 24%).

**Conclusions:**

We did not identify any pattern of previous influenza vaccination effect. Prospective cohort studies documenting influenza infections, vaccinations and vaccine types are needed to understand previous influenza vaccinations' effects.

## 1 INTRODUCTION

Constant evolution of influenza viruses requires possible reformulation of the influenza vaccine every season. In February each year, the World Health Organization (WHO) organises a technical consultation to decide which influenza strains to be included in the Northern Hemisphere seasonal influenza vaccines.^1^

Most groups for whom the seasonal influenza vaccine is recommended may receive a trivalent or quadrivalent inactivated seasonal influenza vaccine annually irrespective of their previous influenza virus infections or influenza vaccination history. In children less than 9 years old, one dose of inactivated influenza vaccine is recommended for children vaccinated in previous season and two doses for those previously unvaccinated.^2^

Several observational studies and meta‐analyses have reported inconsistent results of the effect of previous vaccination on current season influenza vaccine effectiveness (VE).^3^, ^4^, ^5^, ^6^, ^7^, ^8^, ^9^, ^10^, ^11^, ^12^ Some suggest that previous vaccination may reduce the effectiveness of vaccination in the current season.^3^, ^5^, ^6^ Various explanations were proposed. The original “antigenic sin” hypothesis suggests that vaccination primarily boosts pre‐existing antibody responses that cross‐react with the vaccine strain rather than producing a de novo response to the vaccine or infecting strain.^13^ The “antibody block” hypothesis suggests that previously vaccinated individuals do not have the cross‐protective immunity provided by natural infection.^14^, ^15^ According to the “antigenic distance” hypothesis, in seasons when similar strains are included in the subsequent vaccines but are different from the circulating strain, previous vaccination may negatively interfere with current vaccination.^16^ Finally, because the influenza vaccine is recommended to individuals with high‐risk conditions (eg pregnant women, persons with chronic conditions, older adults aged >59 or >64 years), characteristics of individuals repeatedly vaccinated may result in a poorer observed response to the vaccine if we fail to control for negative confounding.^4^

Due to the frequent changes of the genetic and antigenic characteristics of the circulating influenza strains and of those included in the vaccines, data from multiple seasons are needed to measure the potential effects of previous vaccinations and to explore the possible mechanism(s) that may explain such effects.^11^

In this article, using the I‐MOVE primary care multicentre case‐control study (MCCS) data, we present influenza type‐/subtype‐specific VE stratified by previous season vaccination among the target groups for vaccination for each of the 2011/2012 to 2016/2017 seasons. Additionally, using those unvaccinated in both seasons as a reference group, we calculated VE for different combinations of previous/current vaccination among the target population for vaccination (indicator analysis).

Both analyses measure the effect of current vaccination among those not vaccinated in the previous season. The indicator analysis additionally gives information on the potential residual protection of the previous season vaccination and the combined protection of current and previous season vaccination, compared to the reference group. This reference group “unvaccinated in both seasons” may consist of a population that is quite different from the other categories of individuals who have been vaccinated, making controlling for confounding challenging.^11^ Therefore, we present also the stratified analysis, where the stratum of those with previous vaccination indicates the extra protection the current vaccination may have, compared to residual effects of previous vaccination, while controlling adequately for previous vaccination history.

## 2 METHODS

Each season we conducted a primary care–based test‐negative design MCCS. The methods were described previously and are based on the same generic study protocol.^17^

In summary, practitioners from twelve participating sites interviewed a systematic sample of patients consulting for influenza‐like illness (ILI; EU case definition^18^: sudden onset of symptoms and at least one of the following systemic symptoms: fever or feverishness, malaise, headache, myalgia and at least one of the following respiratory symptoms: cough, sore throat, shortness of breath) and collected nasopharyngeal specimens for virological analyses. The data collected include ILI symptoms and date of onset, age, sex, presence of chronic conditions and hospitalisation for the chronic conditions in the previous 12 months.

Vaccination status for current (including date of vaccination and type of vaccine used) and previous season were documented either through patients' self‐report or extracted from practitioners' vaccine registries.

We defined patients as vaccinated in the current season if they had received at least one dose of influenza vaccine more than 14 days before symptom onset. All others with information on current vaccination status were defined as unvaccinated. Patients who received at least one dose of influenza vaccine in the previous season were defined as vaccinated in the previous season. For this analysis, we used the population for which influenza vaccination is recommended every season, as they are likely to be a more homogeneous group in terms of vaccination practices than those for whom vaccination is not recommended. They include older adults (aged over 54, 59 or 64 years depending on study site), individuals with chronic conditions and, where available, other groups for whom the vaccine was recommended in a given country (eg pregnant women, healthcare workers and other professional groups, depending on the study site). We excluded children aged less than 9 years as the definition of their current season vaccination status depends on their previous season vaccination.

Cases were ILI patients testing positive for any type‐/subtype‐specific influenza virus using real‐time reverse‐transcription PCR (RT‐PCR). Controls were those testing negative for all influenza viruses.

Study periods depended upon season‐ and site‐specific influenza virus type/subtype circulation and vaccination campaigns.

We included ILI patients who consulted their practitioner more than 14 days after the start of national or regional seasonal influenza vaccination campaign, who were swabbed less than 8 days after ILI symptom onset and who did not receive influenza antivirals before swabbing. We excluded patients with missing information for current or previous season vaccination status.

We used logistic regression to compute the odds ratio (OR) of being vaccinated in cases and controls. We estimated the type‐/subtype‐adjusted influenza VE as (1 − OR)*100. Study site was modelled as a fixed effect and always included in the crude and adjusted analysis models. We measured VE carrying out a complete case analysis excluding patients with missing values for any of the variables in the model measuring VE. We included age, sex, presence of chronic conditions, pregnancy and obesity, where applicable, and date of symptom onset as a priori confounding variables in the model. All other potential confounders were included in the model if they changed the VE point estimate by 5% (absolute percentage). Age and onset time were modelled as a restricted cubic spline with knots specified according to Harrel.^19^

We measured current season VE for each type/subtype and season. To study the effects of previous vaccination on current season vaccination, we conducted two analyses.

First, we conducted a stratified analysis in which we measured current season VE among individuals vaccinated and unvaccinated in the previous season.

In a second analysis, we used unvaccinated in both seasons as a reference to compare VE between vaccinated in current season only, vaccinated in previous season only and vaccinated in both seasons, using a mutually exclusive indicator variable. We refer to this analysis as the indicator analysis.

For each influenza type/subtype, we used seasons and sites for which I‐MOVE MCCS included at least four cases of this influenza type/subtype per analysed stratum. We deemed sample size too small to attempt an analysis if there were fewer than 50 cases in the pooled study analysis. If the 10 events per variable (EPV) rule was violated,^20^ we carried out a sensitivity analysis using Firth's method of penalised logistic regression to check for small sample bias.

## 3 RESULTS

### 3.1 Recruitment

From season 2011/2012 to 2016/2017, we included between 6 (in 2013/2014) and 12 (in 2015/2016, 2016/2017) sites in the pooled analysis of our multicentre study (Table 1). GPs enrolled 10 831 ILI patients, aged 9 years or older, belonging to the groups targeted for influenza vaccination. Of these, we excluded 368 individuals with missing information on current season vaccination and 405 on previous vaccination. The proportion of individuals excluded due to missing information on current or previous season was 6.9% among cases and 7.3% among controls (Fisher's exact test, *P* = .365). The median age was 63 and 60 years for those with missing and complete information for vaccination status, respectively (*P* < .001).

**Table 1:** Vaccine effectiveness^a^ against influenza type/subtype overall and stratified by previous season vaccination, among the target group for influenza vaccination, aged 9 y or older, I‐MOVE multicentre case‐control study, influenza seasons 2011/12‐2016/2017 (patients with missing data on previous vaccination status excluded)

Type/subtype	Season	Study sites included	Current season VE			Current season VE among those not vaccinated in previous season			Current season VE among those vaccinated in previous season		
			Influenza cases; cases vaccinated/controls; controls vaccinated	VE	95% CI	Influenza cases; cases vaccinated/controls; controls vaccinated	VE	95% CI	Influenza cases; cases vaccinated/controls; controls vaccinated	VE	95% CI
A(H1N1)pdm09	2012/13	DE, FR, ES, IE, PL, PT, RO	172; 27/442; 138	40.0	0.3‐63.9	136; 4/296; 20	62.1	−22.8 to 88.3	36; 23/134; 111	NC	
	2013/14	DE, ES,HU, IE, PT, RO	123; 24/371; 139	56.2	22.3‐75.3	99; 7/244; 28	50.6	−29.1 to 81.1	24; 17/122; 107	**NC**	
	2014/15	DE, ES, FR, HU, IE, IT, PL, RO, SE	171; 31/808; 246	45.4	12.5‐65.9	140; 6/571; 61	62.5	6.3‐85.0	31; 25/230; 180	NC	
	2015/16	DE, ES, FR, HU, HR, IE, IT, NL, PL, PT, RO, SE	454; 105/1239; 390	33.3	9.7‐50.8	345; 10/830; 57	61.3	17.4‐81.9	109; 95/409; 333	−38.4	−178.8 to 31.3
A(H3N2)	2011/12	FR, ES, HU, IE, IT, PT, RO	411; 148/543; 200	25.2	−6.4 to 47.4	273; 21/339; 36	32.7	−35.8 to 66.7	138; 127/204; 164	−68.2	−299.3 to 29.1
	2013/14	DE, ES, HU, IE, PT, RO	151; 48/379; 139	38.2	−1.3 to 62.4	106; 8/252; 28	50.1	−30.9 to 81.0	45; 40/127; 111	NC	
	2014/15	DE, ES, FR, HU, IE, IT, PL, PT, RO, SE	587; 184/918; 292	16.0	−10.0 to 35.9	408; 30/628; 71	47.6	14.2‐68.0	179; 154/289; 220	−21.2	−121.2 to 33.6
	2016/17	DE, ES, FR, HU, HR, IE, IT, NL, PL, PT, RO, SE	1345; 411/1572; 496	21.3	5.7‐34.4	921; 52/1045; 91	45.9	21.1‐62.9	424; 359/527; 405	−18.5	−73.2 to 18.9
B	2012/13	DE, ES, FR, IE, PL, PT, RO	304; 55/468; 145	50.8	26.5‐67.0	243; 9/311; 20	43.0	−33.7 to 75.7	61; 46/125; 97	56.4	−10.0 to 82.7
	2014/15	DE, ES, FR, HU, IE, IT, PL, PT, RO, SE,	320; 65/904; 288	40.2	14.0‐58.4	249; 11/624; 72	60.7	20.3‐80.6	71; 54/269; 207	33.8	−50.3 to 70.8
	2015/16	DE, ES, FR, HR, HU, IE, IT, NL, PL, PT, RO, SE	310; 77/991; 311	19.6	−13.7 to 43.1	220; 9/674; 45	45.9	−18.3 to 75.2	90; 68/314; 263	52.2	1.8‐76.7

DE, Germany; ES, Spain; FR, France; HR, Croatia; HU, Hungary; IE, Ireland; IT, Italy; NL, The Netherlands; PL, Poland; PT, Portugal; RO, Romania; SE, Sweden; VE, vaccine effectiveness; CI, confidence intervals; Casesvacc, cases vaccinated; Controlsvacc, controls vaccinated; NC, not computed since sample size did not allow to include study site as fixed effect.

a VE estimates adjusted by study site, season, age (restricted cubic splines), onset date (restricted cubic splines), chronic condition, sex.

Of the 10 058 ILI individuals with information on current and previous season vaccination status, 19 (0.2%) had influenza virus coinfections reported and were considered in more than one influenza type/subtype analyses. We therefore included 5532 (54.9%) ILI patients negative for all influenza viruses and 941 (9.3%), 2645 (26.2%) and 959 (9.5%) positive for influenza virus A(H1N1)pdm09, A(H3N2) and B, respectively (Table 2).

**Table 2:** Characteristics for influenza A(H1N1)pdm09, A(H3N2) and influenza B cases and controls belonging to the target group for influenza vaccination, aged 9 y or older, I‐MOVE 2011/2012‐2016/2017

Characteristics	Number of test‐negative controls^a^/total n (%)	Number of influenza A(H1N1)pdm09 cases^b^^,^ c/total n (%)	Number of influenza A(H3N2) cases^b^^,^ d/total n (%)	Number of influenza B cases^c^^,^ d/total n (%)
Median age (y)	61.0	54.0	63.0	55.0
Missing	14	5	4	8
Age groups				
9‐14	194/5518 (3.5)	33/936 (3.5)	130/2641 (4.9)	97/951 (10.2)
15‐64	3183/5518 (57.7)	662/936 (70.7)	1333/2641 (50.5)	572/951 (60.1)
≥65	2141/5518 (38.8)	241/936 (25.7)	1178/2641 (44.6)	282/951 (29.7)
Sex				
Female	3249/5515 (58.9)	533/935 (57.0)	1469/2637 (55.7)	511/955 (53.5)
Missing	17	6	8	4
Days between onset of symptoms and swabbing				
0	315/5532 (5.7)	26/941 (2.8)	108/2645 (4.1)	29/959 (3.0)
1	1552/5532 (28.1)	311/941 (33)	874/2645 (33)	245/959 (25.5)
2	1519/5532 (27.5)	312/941 (33.2)	847/2645 (32)	301/959 (31.4)
3	1006/5532 (18.2)	163/941 (17.3)	493/2645 (18.6)	227/959 (23.7)
4‐7	1140/5532 (20.6)	129/941 (13.7)	323/2645 (12.2)	157/959 (16.4)
Current season influenza vaccination				
Not vaccinated or vaccinated <15 d before ILI onset	3771/5532 (68.2)	749/941 (79.6)	1820/2645 (68.8)	759/959 (79.1)
Vaccinated	1761/5532 (31.8)	192/941 (20.4)	825/2645 (31.2)	200/959 (20.9)
Previous season influenza vaccination				
Not vaccinated in current and previous season or vaccinated in current season <15 d before onset	3386/5532 (61.2)	708/941 (75.2)	1708/2645 (64.6)	704/959 (73.4)
Current season vaccination only	324/5532 (5.9)	27/941 (2.9)	116/2645 (4.4)	30/959 (3.1)
Previous season vaccination only	385/5532 (7.0)	41/941 (4.4)	112/2645 (4.2)	55/959 (5.7)
Current and previous season vaccination	1437/5532 (26.0)	165/941 (17.5)	709/2645 (26.8)	170/959 (17.7)
Seasonal vaccination type				
Not vaccinated or vaccinated <15 d before onset	3771/5532 (68.2)	749/941 (79.6)	1820/2645 (68.8)	759/959 (79.1)
Egg‐derived inactivated subunit	521/5532 (9.4)	61/941 (6.5)	244/2645 (9.2)	69/959 (7.2)
Egg‐derived inactivated split virion	638/5532 (11.5)	97/941 (10.3)	309/2645 (11.7)	68/959 (7.1)
Adjuvanted	351/5532 (6.3)	8/941 (0.9)	149/2645 (5.6)	17/959 (1.8)
Cell‐derived inactivated subunit	10/5532 (0.2)	0/941 (0.0)	7/2645 (0.3)	3/959 (0.3)
Live attenuated influenza vaccine	0/5532 (0.0)	0/941 (0.0)	1/2645 (0.0)	0/959 (0.0)
Unknown vaccine type	241/5532 (4.4)	26/941 (2.8)	115/2645 (4.3)	43/959 (4.5)
At least one chronic condition	4105/5436 (75.5)	699/931 (75.1)	1858/2612 (71.1)	700/945 (74.1)
Missing	96	10	33	14
At least one hospitalisation in the previous 12 mo for chronic conditions	352/5075 (6.9)	48/834 (5.8)	128/2462 (5.2)	51/841 (6.1)
Missing	457	107	183	118
Study sites				
Croatia	100/5532 (1.8)	14/941 (1.5)	60/2645 (2.3)	18/959 (1.9)
France	524/5532 (9.5)	126/941 (13.4)	390/2645 (14.7)	168/959 (17.5)
Germany	1397/5532 (25.3)	173/941 (10.3)	505/2645 (19.1)	186/959 (19.4)
Hungary	949/5532 (17.2)	33/941 (2.0)	276/2645 (10.4)	47/959 (4.9)
Ireland	222/5532 (4)	65/941 (4.2)	130/2645 (4.9)	52/959 (5.4)
Italy	476/5532 (8.6)	50/941 (3.3)	222/2645 (8.4)	56/959 (5.8)
Poland	340/5532 (6.1)	70/941 (4.8)	116/2645 (4.4)	59/959 (6.2)
Portugal	402/5532 (7.3)	95/941 (6.7)	175/2645 (6.6)	55/959 (5.7)
Romania	179/5532 (3.2)	74/941 (5.5)	82/2645 (3.1)	53/959 (5.5)
Spain	652/5532 (11.8)	197/941 (9.8)	592/2645 (22.4)	240/959 (25.0)
Sweden	113/5532 (2)	8/941 (0.4)	43/2645 (1.6)	13/959 (1.4)
The Netherlands	178/5532 (3.2)	36/941 (2.0)	54/2645 (2.0)	12/959 (1.3)
Season				
2011‐2012	600/5532 (10.8)	‐	411/2645 (15.5)	‐
2012‐2013	517/5532 (9.3)	181/941 (19.2)	114/2645 (4.3)	313/959 (32.6)
2013‐2014	425/5532 (7.7)	123/941 (13.1)	153/2645 (5.8)	‐
2014‐2015	988/5532 (17.9)	174/941 (18.5)	608/2645 (23.0)	324/959 (33.8)
2015‐2016	1357/5532 (24.5)	463/941 (49.2)	‐	322/959 (33.6)
2016‐2017	1645/5532 (29.7)	‐	1359/2645 (51.4)	‐

a Controls from “any influenza” analysis used.

b One influenza case coinfected for both influenza A(H3N2) and influenza A(H1N1)pdm09 was included in the analysis.

c Nine influenza cases coinfected for both influenza B and influenza A(H1N1)pdm09 were included in the analysis.

d Nine influenza cases coinfected for both influenza B and influenza A(H3N2) were included in the analysis.

The median age was 54, 55, 63 and 61 years among influenza A(H1N1)pdm09, influenza B cases, influenza A(H3N2) cases and controls, respectively.

The proportion of vaccinated with current season vaccine was 32% among controls and 20%, 31% and 21% among influenza A(H1N1)pdm09, A(H3N2) and B cases, respectively (Table 2). The proportion of quadrivalent vaccines used among those with known vaccine brand was <1%.

In each analysis, among cases and controls, less than 6% were vaccinated in the current season only and less than 8% in the previous season only. More than 60% of cases and controls were unvaccinated in both current and previous season and less than 27% were vaccinated in both seasons (Table 2).

The proportion of controls 65 years and older among those vaccinated in both seasons was 64.3% compared to 44.2%, 42.0% and 27.1% among those vaccinated in the previous season only, vaccinated in the current season only and not vaccinated in either season (Table 3).

**Table 3:** Characteristics of test‐negative controls^a^ by vaccination history, among the target population for influenza vaccination, aged 9 y or more, I‐MOVE 2011/2012‐2016/2017

Characteristics	Not vaccinated in either current or previous season	Vaccinated in current season	Vaccinated in previous season only	Vaccinated in both seasons
Median age (y)	56.0	62.0	62.0	69.0
Missing	11	0	0	3
Age groups				
9‐14	153/3375 (4.5)	10/324 (3.1)	8/385 (2.1)	23/1434 (1.6)
15‐64	2307/3375 (68.4)	178/324 (54.9)	207/385 (53.8)	491/1434 (34.2)
≥65	915/3375 (27.1)	136/324 (42.0)	170/385 (44.2)	920/1434 (64.2)
Missing	11	0	0	3
Sex				
Female	1396/3373 (41.4)	128/324 (39.5)	159/385 (41.3)	583/1433 (40.7)
Male	1977/3373 (58.6)	196/324 (60.5)	226/385 (58.7)	850/1433 (59.3)
Missing	13	0	0	4
Days between onset of symptoms and swabbing				
0	212/3386 (6.3)	17/324 (5.2)	17/385 (4.4)	69/1437 (4.8)
1	1021/3386 (30.2)	89/324 (27.5)	105/385 (27.3)	337/1437 (23.5)
2	958/3386 (28.3)	76/324 (23.5)	109/385 (28.3)	376/1437 (26.2)
3	582/3386 (17.2)	59/324 (18.2)	63/385 (16.4)	302/1437 (21.0)
4‐7	613/3386 (18.1)	83/324 (25.6)	91/385 (23.6)	353/1437 (24.6)
At least one chronic condition	2425/3315 (73.2)	253/317 (79.8)	308/380 (81.1)	1119/1424 (78.6)
Missing	71	7	5	13
At least one hospitalisation in the previous 12 mo for chronic conditions	171/3104 (5.5)	20/294 (6.8)	33/346 (9.5)	128/1331 (9.6)
Missing	282	30	39	106
Season				
2011‐2012	351/3386 (10.4)	39/324 (12.0)	42/385 (10.9)	168/1437 (11.7)
2012‐2013	325/3386 (9.6)	24/324 (7.4)	37/385 (9.6)	131/1437 (9.1)
2013‐2014	258/3386 (7.6)	32/324 (9.9)	19/385 (4.9)	116/1437 (8.1)
2014‐2015	607/3386 (17.9)	75/324 (23.1)	72/385 (18.7)	234/1437 (16.3)
2015‐2016	849/3386 (25.1)	59/324 (18.2)	90/385 (23.4)	359/1437 (25.0)
2016‐2017	996/3386 (29.4)	95/324 (29.3)	125/385 (32.5)	429/1437 (29.9)

a Controls from “any influenza” analysis used.

### 3.2 Vaccine effectiveness

We included four influenza seasons for the influenza A(H1N1)pdm09 and A(H3N2) analysis and two for the influenza B analysis (Table 4).

**Table 4:** Predominant influenza strains circulating and influenza strains included in the vaccines in Europe by influenza virus type/subtype and season, 2011/2012‐2016/2017

Type/subtype	Season	Vaccine strains		Main circulating strain(s) in Europe
		Previous season vaccine strain	Current season vaccine strain	
A(H1N1)pdm09	2012/2013	A/California/7/2009 (H1N1)‐like	A/California/7/2009 (H1N1)‐like	A/California/7/2009 (H1N1)‐like
	2013/2014	A/California/7/2009 (H1N1)‐like	A/California/7/2009 (H1N1)‐like	A/California/7/2009 (H1N1)‐like
	2014/2015	A/California/7/2009 (H1N1)‐like	A/California/7/2009 (H1N1)‐like	A/California/7/2009 (H1N1)‐like
	2015/2016	A/California/7/2009 (H1N1)‐like	A/California/7/2009 (H1N1)‐like	A/California/7/2009 (H1N1)‐like
A(H3N2)	2011/2012	A/Perth/16/2009(H3N2)‐like	A/Perth/16/2009(H3N2)‐like	A/Perth/16/2009(H3N2)‐like
				A/Victoria/361/2011(H3N2)like
	2013/2014	A/Victoria/361/2011(H3N2)‐like^a^	A/Texas/50/2012(H3N2)‐like	A/Texas/50/2012(H3N2)‐like
	2014/2015	A/Texas/50/2012(H3N2)‐like	A/Texas/50/2012(H3N2)‐like	A/Switzerland/9715293/2013 (H3N2)‐like
	2016/2017	A/Switzerland/9715293/2013 (H3N2)‐like virus	A/Hong Kong/4801/2014 (H3N2)‐like	A/Hong Kong/4801/2014 (H3N2)‐like
B	2012/2013	B/Brisbane/60/2008‐like (Victoria)	B/Wisconsin/61/2010‐like (Yamagata)	B/Massachusetts/2/2012‐like (Yamagata)
	2014/2015	B/Massachusetts/2/2012‐like (Yamagata)	B/Massachusetts/2/2012‐like (Yamagata)	B/Phuket/3073/2013‐like (Yamagata)
	2015/2016	B/Massachusetts/2/2012‐like (Yamagata)	B/Phuket/3073/2013‐like (Yamagata)	B/Brisbane/60/2008‐like(Victoria)

a An egg‐adaptative mutation in the egg‐propagated A/Victoria/361/2011 modified the antigenic characteristics of the vaccine strain that resulted in a strain antigenically different from the circulating Texas viruses.

#### 3.2.1 Current season VE

The current season adjusted VE against influenza A(H1N1)pdm09 ranged from 33% (95% CI: 10‐51) in 2015/2016 to 56% (95% CI: 22‐75) in 2013/2014.

Current season VE against influenza A(H3N2) ranged from 16% (95% CI: −10 to 36) in 2014/2015 to 38% in 2013/2014 (95% CI: −1 to 62), and against B, it ranged from 20% (95% CI: −14 to 43) in 2015/216 to 51% (95% CI: 27‐68) in 2012/2013 (Table 1).

#### 3.2.2 Stratified analysis, current season VE according to previous season vaccination

##### Influenza A(H1N1)pdm09

Among those not vaccinated in the previous season, the current season adjusted VE ranged from 51% (95% CI: −29 to 81) in 2013/2014 to 63% (95% CI: 6‐85) in 2015/2016. We could only compute current season adjusted VE among those vaccinated in the previous season for one season due to the high proportion of individuals vaccinated in both current and previous season. In the 2015/2016 season, the VE against A(H1N1)pdm09 among those vaccinated in the previous season was −38% (95% CI: −179 to 31) (Table 1).

##### Influenza A(H3N2)

We could compute VE stratified by previous vaccination status in three of the four seasons. In these three seasons, current season VE point estimate among those vaccinated in previous season was negative (−68%, −21% and −19% in 2011/2012, 2014/2015, 2016/2017, respectively) and lower than among those unvaccinated in previous season (33%, 48% and 46% in 2011/2012, 2014/2015 and 2016/2017, respectively) (Table 1).

##### Influenza B

Among those not vaccinated in previous season, current season adjusted VE ranged from 43% (95% CI: −34 to 76) in 2012/2013 to 61% (95% CI: 20‐81) in 2014/2015. The current season VE among those vaccinated in the previous season ranged from 34% to 48% (Table 1).

#### 3.2.3 Indicator analysis, using those unvaccinated in both seasons as reference

##### Influenza A(H1N1)pdm09

In 2013/2014, the VE point estimate for vaccination in both current and previous seasons was higher than that for vaccination in the current season only (Table 5). In the other three seasons, VE point estimates for vaccination in both seasons were lower than those for vaccination in current season only. VE point estimates for vaccination in previous season only were 35% in 2014/2015 and 47% in 2015/2016 and negative in both 2012/2013 and 2013/2014 (Table 5).

**Table 5:** Vaccine effectiveness^a^ for vaccination in current season only, previous season only or both seasons among the target population for influenza vaccination, aged 9 y or more, by influenza type/subtype and influenza strains circulating and included in the current and previous season vaccine, I‐MOVE 2011/2012‐2016/2017

Type/subtype	Season	Vaccinated current season only			Vaccinated previous season only			Vaccinated both seasons		
		Cases (% of all cases)/controls (% of all controls)	VE	95% CI	Cases (% of all cases)/controls (% of all controls)	VE	95% CI	Cases (% of all cases)/controls (% of all controls)	VE	95% CI
A(H1N1)pdm09	2012/2013	4 (2.3)/20 (4.5)	59.7	−28.9 to 87.4	13 (7.6)/28 (6.3)	−7.9	−135.0 to 50.4	23 (13.4)/118 (26.7)	33.8	−15.4 to 62.1
	2013/2014	7 (5.7)/28 (7.5)	40.3	−51.7 to 76.5	7 (5.7)/16 (4.3)	−7.8	−199.4 to 61.2	17 (13.8)/111 (29.9)	60.6	24.3‐79.5
	2014/2015	6 (3.5)/61 (7.5)	60.7	2.7‐84.1	6 (3.5)/52 (6.4)	35.1	−63.0 to 74.2	25 (14.6)/185 (22.9)	42.4	3.2‐65.7
	2015/2016	10 (2.2)/57 (4.6)	60.4	15.9‐81.3	14 (3.1)/76 (6.1)	46.6	−2.0 to 72.1	95 (20.9)/333 (26.9)	31.8	5.7‐50.6
A(H3N2)	2011/2012	21 (5.1)/36 (6.6)	37.8	−24.9 to 69.1	11 (2.7)/40 (7.4)	59.8	11.2‐81.8	127 (30.9)/164 (30.2)	29.4	−3.2 to 51.7
	2013/2014	8 (5.3)/28 (7.4)	44.6	−38.8 to 77.9	5 (3.3)/16 (4.2)	28.7	−133.3 to 78.2	40 (26.5)/111 (29.3)	39.1	−4.1 to 64.4
	2014/2015	30 (5.1)/71 (7.7)	47.5	14.8‐67.6	25 (4.3)/69 (7.5)	15.1	−45.7 to 50.5	154 (26.2)/221 (24.1)	5.2	−27.5 to 29.5
	2016/2017	52 (3.9)/91 (5.8)	45.8	21.5‐62.6	65 (4.8)/122 (7.8)	30.8	2.1‐51.1	359 (26.7)/405 (25.8)	18.8	1.0‐33.3
B	2012/2013	9 (3.0)/20 (4.3)	39.4	−42.7 to 74.3	15 (4.9)/32 (6.8)	4.0	−93.1 to 52.3	46 (15.1)/125 (26.7)	53.0	27.5‐69.5
	2014/2015	11 (3.4)/72 (8.0)	59.1	17.1‐79.8	17 (5.3)/64 (7.1)	−6.2	−103.3 to 44.5	54 (16.9)/216 (23.9)	32.8	−0.3 to 54.9
	2015/2016	9 (2.9)/45 (4.5)	46.6	−17.0 to 75.7	22 (7.1)/51 (5.1)	−37.5	−152.9 to 25.3	68 (21.9)/266 (26.8)	9.4	−31.7 to 37.6

VE, vaccine effectiveness; CI, confidence interval.

a VE estimates adjusted by study site, season, age (restricted cubic splines), onset date (restricted cubic splines), chronic condition, sex.

##### Influenza A(H3N2)

In 2013/2014, 2014/2015 and 2016/2017, VE point estimates for vaccination in both current and previous seasons were lower than those for vaccination in current season only (Table 5). In 2011/2012, VE point estimates for those two groups were similar (less than 9% absolute difference). VE point estimates, for vaccination in previous season only, ranged from 15% to 60% (Table 5).

##### Influenza B

In 2012/2013, the VE point estimate for vaccination in both current and previous seasons was higher than that for vaccination in current season only. In 2014/2015 and 2015/2016, VE point estimates for vaccination in both seasons were lower than those for vaccination in the current season only. VE point estimates for vaccination in the previous season only were 4% in 2012/2013 and negative for the other two seasons (Table 5).

#### 3.2.4 Sensitivity analysis

Where the EPV was <10, penalised and standard logistic regression VE estimates did not differ by more than 6.5% absolute, with an average of 3.4%, for both the stratified and indicator analyses, indicating little bias due to sparse data.

## 4 DISCUSSION

Our results suggest that, based on the stratified analysis results, previous vaccination may have an effect on current season VE among the population targeted for influenza vaccination. Despite low precision, the current season VE point estimates against influenza A(H1N1)pdm09 in the 2015/2016 season and against influenza A(H3N2) in the seasons where calculable (2011/2012, 2014/2015, 2016/2017) were lower among those vaccinated in the previous season than among those not vaccinated in previous season. These VE estimates were less than 0%, suggesting a negative effect of previous vaccination. This was not seen against influenza B, where the VE among those previously vaccinated was positive, indicating an effect of current vaccination additionally to any residual protection from previous vaccination. However, precision is low for most of these analyses and the current VE among those vaccinated previously was not possible to compute, so we cannot draw conclusions on a consistent or common pattern for the three influenza types/subtypes.

Individuals unvaccinated in both seasons are at a higher risk of laboratory‐confirmed influenza than in any vaccination scenario (current season only, last season only, both seasons). This would suggest that, even if the effect can be limited in some seasons, whatever the past vaccination scenario, being vaccinated in a season is always beneficial.

In the 2011/2012 and 2016/2017 influenza A(H3N2) seasons and in the 2015/2016 influenza A(H1N1)pdm09 analysis, the VE point estimates and confidence intervals among those vaccinated only in the previous season suggest a protective effect of previous season vaccine for those subtypes. This is consistent with the duration of protection observed for A(H1N1)pdm09, but contradictory to the short duration of protection for A(H3N2).^21^

Our study has several limitations. Even though our analysis is restricted to the target group for vaccination, the vaccination coverage is still low (6%, 7% and 26% of controls vaccinated in current season only, in previous season only and in both seasons, respectively). This results in a low precision and high variability of the results.

Past infections and natural immunity play an important role in the protection against circulating influenza strains 22, 23 and may strengthen the response to subsequent vaccines.^24^ Measuring immunity conferred by past infection is challenging. An immunological study suggests that the response to inactivated influenza vaccines is lower among individuals previously vaccinated than among individuals with previous natural influenza A(H1N1)pdm09 virus infection.^25^ In a hospital‐based study in Japan, a negative effect of previous vaccination on current season vaccine effectiveness was observed in individuals not infected but not in individuals infected with influenza A virus in the previous season.^8^ Individuals in the same age group may have had similar exposure to previous circulating influenza viruses, resulting in a similar influenza antibody landscape.^26^, ^27^, ^28^ In our study, the low vaccination coverage and number of cases in some age groups did not allow for estimation of results by age group.

We only collected information on the effect of one previous season vaccination and were not able to take into account the role of repeated previous vaccinations. In a study using eight seasons' pooled data and five previous years of historical vaccination, VE was similar for those vaccinated in the current season and those vaccinated in current and previous seasons.^4^ However, VE was lower for current season in the group of individuals vaccinated at least in two of five seasons than in individuals never vaccinated in the five seasons.

We documented the vaccine type for the current season but not for the previous season. Studies suggest that the type of vaccine received in current or previous season may influence the effect of previous vaccination in current season VE.^4^, ^7^ In our study, four types of vaccines were used: egg‐derived inactivated subunit, egg‐derived inactivated split virion, cell‐derived inactivated subunit and adjuvanted vaccines (adjuvant used: squalene (MF59) or aluminium phosphate gel). We could not stratify VE results by type of current and previous vaccines.

While the test‐negative design attempts to control for bias due to differential healthcare‐seeking behaviours,^29^ the study is observational and subject to the usual limitations, in particular adequately controlling for confounding.

### 4.1 Results interpretation

#### 4.1.1 Influenza A(H1N1)pdm09

The stratified results in the 2015/2016 season suggested that the effectiveness of current season vaccination against influenza A(H1N1)pdm09 was lower among those vaccinated in the previous season. When using unvaccinated in both seasons as reference, VE point estimates for current season vaccination only were higher than for both seasons vaccination in three of the four seasons. The positive VE of previous season vaccination only in the 2014/2015 and 2015/2016 season analysis suggests a potential residual effect of vaccination against influenza A(H1N1)pdm09 in these seasons. This observation is in line with two recent meta‐analysis^11^, ^30^ and with the stable influenza A(H1N1)pdm09 VE by time since vaccination observed in the I‐MOVE MCCS from 2010/2011 to 2015/2016.^21^

#### 4.1.2 Influenza A(H3N2)

We observed that in all seasons, A(H3N2) VE point estimates were lower among those vaccinated in the previous season than in those unvaccinated in previous season.

Using unvaccinated in both seasons as reference, VE of various vaccination scenarios varied by season. In 2011/2012 and 2013/2014, VE point estimates against influenza A(H3N2) were similar for current season vaccination only and both seasons' vaccination. In 2011/2012, previous and current season vaccine strains were antigenically similar but mismatched to one of the cocirculating strains. In 2013/2014, the circulating strains were antigenically similar to the vaccine strain but different to the 2012/2013 vaccine strain that had egg‐adapted amino acid changes negatively impacting its antigenicity.^31^

In 2014/2015, VE against influenza A(H3N2) was much lower among those vaccinated in 2013/2014 and 2014/2015 than among those vaccinated only in 2014/2015. The influenza A(H3N2) vaccine strains in 2013/2014 and 2014/2015 were homologous but antigenically different from the drifted virus circulating in 2014/2015. Even if the precision is low, our results are in accordance with the antigenic distance hypothesis.^16^ In the 2014/2015 VE results from Canada^5^, ^10^ and from I‐MOVE MCCS (not restricted to the target population),^6^ the VE point estimate among those vaccinated in both 2013/2014 and 2014/2015 was negative, suggesting that those vaccinated in both seasons were at higher risk of influenza A(H3N2) medically attended influenza than those vaccinated only in 2014/2015. In the current I‐MOVE MCCS analysis restricted to the vaccine target population, the VE point estimate for those vaccinated in both seasons was positive but lower than the VE of those vaccinated only in 2014/2015.

In 2016/2017, the VE point estimate was also lower for those vaccinated in both 2016/2017 and 2015/2016 seasons than in those vaccinated only in 2016/2017. The antigenic hypothesis may not explain this observation as the A(H3N2) circulating strain and the strains included in the 2016/2017 and 2015/2016 vaccines were considered antigenically similar. However, it is worth noting that in the 2016/2017 season, the A(H3N2) viruses circulating in Europe underwent considerable genetic changes with new subgroups diversifying from the vaccine strains.^32^

#### 4.1.3 Influenza B

The low precision of the VE against influenza B did not allow us to identify any pattern of the effect of previous vaccination. However VE point estimates for vaccination in current season only were always positive and VE point estimates for vaccination in previous season only were low or negative. We could not measure VE by influenza B lineage which limits interpretation.

## 5 CONCLUSION

We cannot exclude that being vaccinated in the previous season may reduce the VE of the current season vaccine, in particular for A(H3N2). However, the positive VE results among those vaccinated in current season only and those vaccinated in both seasons in this study suggest that being vaccinated in a given season is always beneficial.

Despite a growing sample size in I‐MOVE MCCS, results are still imprecise due to low vaccination coverage and to the strong association between being vaccinated in current season and being vaccinated in previous season. The results of the six seasons can only contribute to test hypotheses that try to understand the effect of previous vaccination on influenza VE.

Influenza vaccine effectiveness depends on complex interactions between host (eg natural infection vs vaccination acquired immunity), agent and environmental factors.^32^ Optimum measurement and interpretation of influenza seasonal VE would require taking into account many parameters including subtypes, clade, subclades, age, time since vaccination, chronic conditions and treatment, duration of protection, vaccine types and brands, natural immunity and previous vaccinations.

So far, the studies presenting the effect of previous season influenza vaccinations neither collected all this information nor were powered enough to provide precise results. The modifying or confounding effect of those parameters would no longer be relevant in terms of public health if the influenza vaccine had a high effectiveness.

Only well‐powered, population‐based, long prospective studies would allow understanding the immunological response to influenza vaccinations and infections. They could document influenza infections, vaccinations and type of vaccines used, cellular and humoural immune status before and during each season in vaccinated and unvaccinated, infected and not. A European mechanism of funding such large studies is needed to ensure powerful studies conducted independently from funding sources.

In the context of universal influenza vaccine development, the question is whether results of these proposed prospective studies would be available before the next generation of vaccines is accessible.

## AUTHOR CONTRIBUTIONS

**EpiConcept**: Marta Valenciano: coordination of I‐MOVE network, study design, interpretation of results, manuscript writing. Esther Kissling: study design, analysis of data, interpretation of results, contribution to manuscript writing. Alain Moren: study design, interpretation of results, contribution to manuscript writing. Study sites: data collection, data validation, results interpretation, review of manuscript. Laboratories: virological analysis, interpretation of results. ECDC: Kari Johansen, Pasi Penttinen: study design, interpretation of results, review of manuscript. The I‐MOVE/I‐MOVE+ study team is very grateful to all patients, general practitioners, paediatricians, hospital teams, laboratory teams, regional epidemiologists who have contributed to the study. Thank you to Marc Rondy, EpiConcept, for his contribution and review of previous versions of the manuscript.

## CONFLICT OF INTEREST

None.

## APPENDIX 1

### List I‐MOVE multicentre primary care case‐control team study team

#### EPICONCEPT, FRANCE

Esther Kissling, EpiConcept; Alain Moren, EpiConcept; Marta Valenciano, EpiConcept.

#### CROATIA

Bernard Kaic, Croatian Institute of Public Health; Sanja Kurecic‐Filipovic, Croatian Institute of Public Health; Iva Pem‐Novosel, Croatian Institute of Public Health; Zvjezdana Lovric, Croatian Institute of Public Health.

#### FRANCE, SENTINELLES

Alessandra Falchi, EA7310, Laboratoire de Virologie, Université de Corse‐Inserm, F‐20250, Corse; Ana‐Maria Vilcu, Sorbonne Universités, UPMC Univ Paris 06, INSERM, Institut Pierre Louis d'épidémiologie et de Santé Publique (IPLESP UMRS 1136); Cécile Souty, Sorbonne Universités, UPMC Univ Paris 06, INSERM, Institut Pierre Louis d'épidémiologie et de Santé Publique (IPLESP UMRS 1136); Thierry Blanchon, Sorbonne Universités, UPMC Univ Paris 06, INSERM, Institut Pierre Louis d'épidémiologie et de Santé Publique.

#### FRANCE, CENTER OF THE NATIONAL REFERENCE CENTER FOR INFLUENZA VIRUSES

Sylvie Behillil, Coordinating Center of the National Reference Center for influenza viruses, Unit of Molecular Genetics of RNA Viruses, Institut Pasteur, UMR3569 CNRS, University Paris Diderot Sorbonne Paris Cité, Institut Pasteur; Vincent Enouf, Coordinating Center of the National Reference Center for influenza viruses, Unit of Molecular Genetics of RNA Viruses, Institut Pasteur, UMR3569 CNRS, University Paris Diderot Sorbonne Paris Cité, Institut Pasteur; Sylvie van der Werf, Coordinating Center of the National Reference Center for influenza viruses, Unit of Molecular Genetics of RNA Viruses, Institut Pasteur, UMR3569 CNRS, University Paris Diderot Sorbonne Paris Cité, Institut Pasteur.

#### FRANCE GROG STUDY

Isabelle Daviaud, Réseau des GROG & Open Rome; Anne Mosnier, Réseau des GROG & Open Rome; Jean Marie Cohen, Réseau des GROG & Open Rome; Martine Valette, National Reference Center for influenza viruses (southern France); Laboratory of virology, Hospices Civils de Lyon; Bruno Lina, National Reference Center for influenza viruses (southern France), Laboratory of virology, Hospices Civils de Lyon, Univ Lyon, Virpath, CIRI, INSERM U1111, CNRS, ENS, Université Claude Bernard Lyon 1, Lyon, France.

#### GERMANY

Annicka Reuss, Department for Infectious Disease Epidemiology, Robert Koch Institute; Brunhilde Schweiger, National Reference Center for Influenza, Robert Koch Institute; Ute Preuß, Department for Infectious Disease Epidemiology, Robert Koch Institute; Silke Buda, Department for Infectious Disease Epidemiology, Robert Koch Institute; Kerstin Prahm, Department for Infectious Disease Epidemiology, Robert Koch Institute; Marianne Wedde, National Reference Center for Influenza, Robert Koch Institute; Alla Heider, National Reference Center for Influenza, Robert Koch Institute; Maria Martin, National Reference Center for Influenza, Robert Koch Institute; Barbara Biere, National Reference Center for Influenza, Robert Koch Institute.

#### HUNGARY

Judit Krisztina Horváth, National Center for Epidemiology, Department of Disease Prevention and Surveillance; Annamária Ferenczi, National Center for Epidemiology, Department of Disease Prevention and Surveillance; Beatrix Oroszi, National Center for Epidemiology; Zita Vizler, Office of the Chief Medical Officer; Éva Hercegh, National Center for Epidemiology, Influenza Virus Laboratory; Bálint Szalai, National Center for Epidemiology, Influenza Virus Laboratory.

#### IRELAND

Joan O'Donnell, HSE‐Health Protection Surveillance Centre; Lisa Domegan, HSE‐Health Protection Surveillance Centre; Anita Kelly, HSE‐Health Protection Surveillance Centre; Michael Joyce, Irish College of General Practitioners; Claire Collins, Irish College of General Practitioners; Cillian de Gascun, National Virus Reference Laboratory, University College Dublin; Jeff Connell, National Virus Reference Laboratory, University College Dublin; Grainne Tuite, National Virus Reference Laboratory, University College Dublin; Margaret Duffy, National Virus Reference Laboratory, University College Dublin; Joanne Moran, National Virus Reference Laboratory, University College Dublin; Bridget Hogg, National Virus Reference Laboratory, University College Dublin; Allison Waters, National Virus Reference Laboratory, University College Dublin; Linda Dunford, National Virus Reference Laboratory, University College Dublin.

#### ITALY

Caterina Rizzo, Department of Infectious Diseases Istituto Superiore di Sanità; Antonino Bella, Department of Infectious Diseases Istituto Superiore di Sanità; Valeria Alfonsi, Department of Infectious Diseases Istituto Superiore di Sanità; Maria Rita Castrucci, National Influenza Center Istituto, Superiore di Sanità; Simona Puzelli, National Influenza Center, Istituto Superiore di Sanità; Annapina Palmieri, National Influenza Center, Istituto Superiore di Sanità; Elena Pariani, Department of Biomedical Sciences for Health, University of Milan; Danilo Cereda, Regional Health Authority, Lombardy Region; Cinzia Germinario, Department of Biomedical Science and Human Oncology, Aldo Moro University of Bari; Maria Chironna, Department of Biomedical Science and Human Oncology, Aldo Moro University of Bari, Bari, University of Bari; Donatella Tiberti, Regional Reference Service for Infectious Diseases Epidemiology, ASL AL, Alessandria; Valeria Ghisetti, Department of Infectious Diseases, Amedeo di Savoia Hospital, Turin; Maria Grazia Pascucci, Regional Health Authority, Emilia‐Romagna Region; Paola Affanni, Department of Medicine and Surgery, University of Parma; Barbara Camilloni, Department of Experimental Medicine, University of Perugia; Pierlanfranco D'Agaro, Department of Medicine, Surgery and Public Health, University of Trieste; Tolinda Gallo, Regional Health Authority, Friuli Venezia Giulia Region.

#### POLAND

Iwona Paradowska‐Stankiewicz, National Institute of Public Health‐National Institute of Hygiene, Warsaw; Monika Korczyńska, National Institute of Public Health‐National Institute of Hygiene, Warsaw; Lidia Brydak, National Institute of Public Health‐National Institute of Hygiene, Warsaw; Justyna Rogalska, Ministry of Interior and Administrative, Warsaw.

#### PORTUGAL

Baltazar Nunes, Departamento de Epidemiologia, Instituto Nacional de Saúde Dr. Ricardo Jorge; Ausenda Machado, Departamento de Epidemiologia, Instituto Nacional de Saúde Dr. Ricardo Jorge; Ana Paula Rodrigues, Departamento de Epidemiologia, Instituto Nacional de Saúde Dr. Ricardo Jorge; Verónica Gomez, Departamento de Epidemiologia, Instituto Nacional de Saúde Dr. Ricardo Jorge; Raquel Guiomar, Departamento de Doenças Infeciosas, Instituto Nacional de Saúde Dr. Ricardo Jorge; Pedro Pechirra, Departamento de Doenças Infeciosas, Instituto Nacional de Saúde Dr. Ricardo Jorge; Paula Cristóvão, Departamento de Doenças Infeciosas, Instituto Nacional de Saúde Dr. Ricardo Jorge; Patrícia Conde, Departamento de Doenças Infeciosas, Instituto Nacional de Saúde Dr. Ricardo Jorge; Inês Costa, Departamento de Doenças Infeciosas, Instituto Nacional de Saúde Dr. Ricardo Jorge.

#### ROMANIA

Daniela Pitigoi, University of Medicine and Pharmacy Carol Davila, National Institute for Research Cantacuzino, Bucharest, Romania; Alina Elena Ivanciuc, National Institute for Research Cantacuzino, Faculty of Biology, Bucharest University; Emilia Lupulescu, National Institute for Research Cantacuzino; Mihaela Lazar, National Institute for Research Cantacuzino; Maria Elena Mihai, National Institute for Research Cantacuzino; Carmen Maria Cherciu, National Institute for Research Cantacuzino.

#### SPAIN

Amparo Larrauri, National Centre of Epidemiology, Institute of Health Carlos III CIBER  Epidemiología y Salud Pública (CIBERESP); Alin Gherasim, National Centre of Epidemiology, Institute of Health Carlos III, CIBER  Epidemiología y Salud Pública (CIBERESP); Silvia Jiménez‐Jorge, National Centre of Epidemiology, Institute of Health Carlos III, CIBER Epidemiología y Salud Pública (CIBERESP); Camelia Savulescu, National Centre of Epidemiology, Institute of Health Carlos III, CIBER Epidemiología y Salud Pública (CIBERESP); Francisco Pozo, National Centre for Microbiology, National Influenza Reference Laboratory, WHO‐National Influenza Centre, Institute of Health Carlos III; I Casas, National Centre for Microbiology, National Influenza Reference Laboratory, WHO‐National Influenza Centre, Institute of Health Carlos III; Jesús Castilla, Instituto de Salud Pública de Navarra, IdiSNA, Pamplona, CIBER  Epidemiología y Salud Pública (CIBERESP); Manuel García Cenoz, Instituto de Salud Pública de Navarra, IdiSNA, Pamplona, CIBER Epidemiología y Salud Pública (CIBERESP); Itziar Casado, Instituto de Salud Pública de Navarra, IdiSNA, Pamplona, CIBER  Epidemiología y Salud Pública (CIBERESP); Fernando Gonzalez Carril, Departamento de Salud, Gobierno del País Vasco; Jone M. Altzibar, Subdirección de Salud Pública de Guipuzkoa, País Vasco. CIBERESP; Carmen Quiñones, Servicio de Epidemiología, Subdirección de Salud Pública de La Rioja; Jaume Giménez, Servicio de Epidemiología, Dirección General de Salut Pública, Palma de Mallorca, Baleares, CIBERESP; Daniel Castrillejo, Servicio de Epidemiología. DGSC, Ciudad Autónoma de Melilla; Tomás Vega, Dirección General de Salud Pública e Investigación, Desarrollo e Innovación, Consejería de Sanidad de Castilla y León; Virtudes Gallardo, Servicio de Epidemiología y Salud Laboral, Secretaría General de Salud Pública y Participación de la Consejería de Salud de Andalucía; Nuria Torner, Servicio de Vigilancia Epidemiológica, Dirección General de Salud Pública, Cataluña, CIBER Epidemiología y Salud Pública (CIBERESP); Julián M. Ramos, Dirección General de Salud Pública, Servicio Extremeño de Salud, Junta de Extremadura.

#### SWEDEN

Mia Brytting, the Public Health Agency of Sweden; Katherina Zakikhany, the Public Health Agency of Sweden.

#### THE NETHERLANDS

Adam Meijer, National Institute for Public Health and the Environment (RIVM); Marit de Lange, National Institute for Public Health and the Environment (RIVM); Frederika Dijkstra, National Institute for Public Health and the Environment (RIVM); Wim van der Hoek, National Institute for Public Health and the Environment (RIVM); Pieter Overduin, National Institute for Public Health and the Environment (RIVM); Anne Teirlinck, National Institute for Public Health and the Environment (RIVM); Gé Donker, Netherlands Institute for Health Services Research (NIVEL).

#### ECDC

Kari Johansen, European Centre for Disease Prevention and Control; Pasi Penttinen, European Centre for Disease Prevention and Control.
